# cAMP controls cytosolic Ca^2+ ^levels in *Dictyostelium discoideum*

**DOI:** 10.1186/1471-2121-6-12

**Published:** 2005-03-07

**Authors:** Daniel F Lusche, Karen Bezares-Roder, Kathrin Happle, Christina Schlatterer

**Affiliations:** 1 Faculty for Biology, University of Konstanz, 78457 Konstanz, Germany

## Abstract

**Background:**

Differentiating *Dictyostelium discoideum *amoebae respond upon cAMP-stimulation with an increase in the cytosolic free Ca^2+ ^concentration ([Ca^2+^]_i_) that is composed of liberation of stored Ca^2+ ^and extracellular Ca^2+^-influx. In this study we investigated whether intracellular cAMP is involved in the control of [Ca^2+^]_i_.

**Results:**

We analyzed Ca^2+^-fluxes in a mutant that is devoid of the main cAMP-phosphodiesterase (PDE) RegA and displays an altered cAMP metabolism. In suspensions of developing cells cAMP-activated influx of extracellular Ca^2+ ^was reduced as compared to wild type. Yet, single cell [Ca^2+^]_i_-imaging of *regA*^- ^amoebae revealed a cAMP-induced [Ca^2+^]_i _increase even in the absence of extracellular Ca^2+^. The cytosolic presence of the cAMP PDE inhibitor 3-isobutyl-1-methylxanthine (IBMX) induced elevated basal [Ca^2+^]_i _in both, mutant and wild type cells. Under this condition wild type cells displayed cAMP-activated [Ca^2+^]_i_-transients also in nominally Ca^2+^-free medium. In the mutant strain the amplitude of light scattering oscillations and of accompanying cAMP oscillations were strongly reduced to almost basal levels. In addition, chemotactic performance during challenge with a cAMP-filled glass capillary was altered by EGTA-incubation. Cells were more sensitive to EGTA treatment than wild type: already at 2 mM EGTA only small pseudopods were extended and chemotactic speed was reduced.

**Conclusion:**

We conclude that there is a link between the second messengers cAMP and Ca^2+^. cAMP-dependent protein kinase (PKA) could provide for this link as a membrane-permeable PKA-activator also increased basal [Ca^2+^]_i _of *regA*^- ^cells. Intracellular cAMP levels control [Ca^2+^]_i _by regulating Ca^2+^-fluxes of stores which in turn affect Ca^2+^-influx, light scattering oscillations and chemotactic performance.

## Background

Starving *Dictyostelium discoideum *amoebae form a multicellular organism by chemotactic aggregation. The signaling molecule that mediates aggregation and development is cAMP. Aggregation proceeds in a rhythmic fashion; cAMP is secreted periodically by cells in the center of the aggregate. Cells in the neighbourhood respond by an oriented inward movement and secrete cAMP themselves to relay the signal. In cell suspensions periodic synthesis and release of cAMP leads to rhythmic shape changes that cause alterations in light transmittance and spike-shaped and sinusoidal light scattering oscillations [1]. The marked rhythmic behaviour of the cell population is also apparent by oscillations of other parameters, e.g. extracellular concentrations of Ca^2+^, K^+ ^or H^+ ^(for review see [2]). Recently, changes in [Ca^2+^]_i _were postulated to comprise the (or at least a part of the) master oscillator controlling oscillation patterns [3,4]. A short [Ca^2+^]_i_-transient induced by addition of CaCl_2 _or calmodulin antagonists alters light scattering oscillations and can even reset the oscillation phase [3]. The height of the [Ca^2+^]_i_-increase determines whether light scattering and the accompanying cAMP oscillations are abolished or augmented: large [Ca^2+^]_i_-transients inhibit cAMP and light scattering oscillations [3] whereas small [Ca^2+^]_i_-elevations enhance oscillations of both parameters [4]. From these experiments it was concluded that Ca^2+ ^exerts a dual control over the production of the first messenger cAMP (for a detailed model see [4]). cAMP controls its own synthesis as binding of the agonist to cell surface receptors induces a transient [Ca^2+^]_i_-elevation [5-7]. However, until now the question as to whether there is an interaction between cAMP acting intracellularly as second messenger and [Ca^2+^]_i _in *D. discoideum *has not been resolved. In other cell systems such as nerve cells crosstalk between the cAMP and the Ca^2+ ^signaling pathway exists that is necessary to generate oscillations of both parameters [8].

In order to gain insight into a possible connection between intracellular cAMP and [Ca^2+^]_i _we used a mutant defective in the phosphodiesterase RegA. RegA is one out of two cAMP-specific phosphodiesterases (for an overview of classes of PDEs in *Dictyostelium *see [9]) that is inhibited by IBMX and comprises part of an eukaryotic phospho-relay system [10,11]. *RegA*^- ^mutants are rapid developers; their differentiation is shifted towards the stalk pathway [12,13]. Chemotactic migration is characterized by an increased frequency of lateral pseudopod extension as compared to wild type amoebae [14]. We found that the mutant displayed an altered [Ca^2+^]_i_-response pattern upon stimulation with cAMP with an augmentation of Ca^2+^-release from stores and a concomitant decrease of extracellular Ca^2+^-entry. Light scattering oscillations and the underlying cAMP oscillations were drastically reduced in *regA*^- ^cells. Chemotaxis was influenced by the extracellular presence of EGTA. We conclude that indeed, intracellular cAMP signaling and the regulation of [Ca^2+^]_i _are linked at the level of Ca^2+^-storage compartments.

## Results

### Extracellular and intracellular [Ca^2+^]-recordings

**Figure 1:**
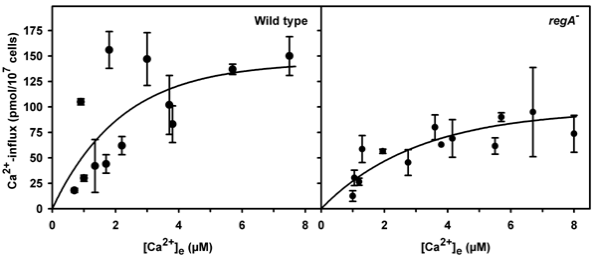
Ca^2+^-influx after cAMP stimulation is reduced in *regA*^- ^cells. The amount of influx (pmol/10^7 ^cells) after addition of 1 μM cAMP is plotted versus extracellular [Ca^2+^]. Average influx was higher in wild type than in *regA*^- ^amoebae (mean ± s.d. from at least 6 determinations in 3 independent experiments each).

To test whether the absence of the main cAMP-specific phosphodiesterase affects regulation of [Ca^2+^]_i _we analyzed extracellular Ca^2+^-fluxes in cell suspensions and studied [Ca^2+^]_i _in single amoebae. cAMP-induced Ca^2+^-influx in suspensions of *regA*^- ^cells occurred with a similar time course as in wild type. Yet, influx was reduced by approximately 40% (Fig. 1). The loss of RegA should lead to an altered cAMP metabolism. Indeed, the basal total amount of cAMP was increased fourfold (13 ± 3 pmol/10^7 ^*regA*^- ^cells; mean ± s.e.m. of 16 determinations in 7 independent experiments vs. 2.8 ± 0.3 pmol/10^7 ^wild type cells; mean ± s.e.m. of 11 determinations in 6 independent experiments). Addition of the PDE inhibitor IBMX (up to 200 μM) to wild type cells affected neither the amount nor the characteristics of cAMP-activated extracellular Ca^2+^-fluxes.

**Figure 2:**
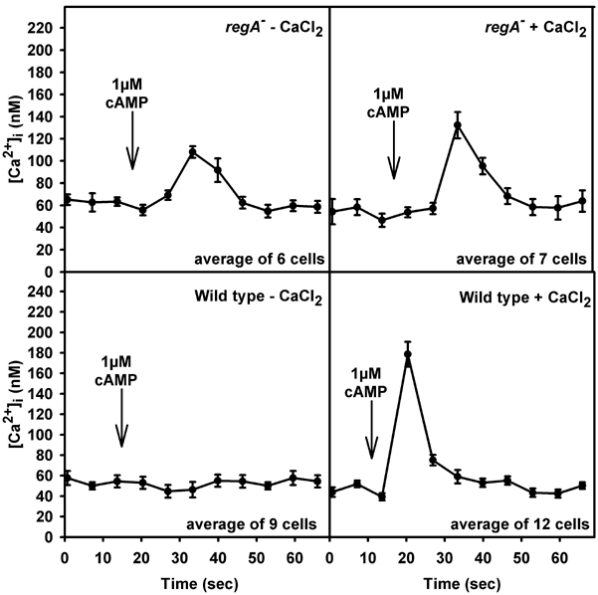
Measurement of cAMP activated [Ca^2+^]_i_-changes in wild type and mutant amoebae. Cells were stimulated with 1 μM cAMP in the presence or absence of 1 mM external CaCl_2_. In wild type amoebae a [Ca^2+^]_i_-transient was observed in the presence of external Ca^2+^. The graph shows the average increase (mean ± s.e.m.).

**Figure 3:**
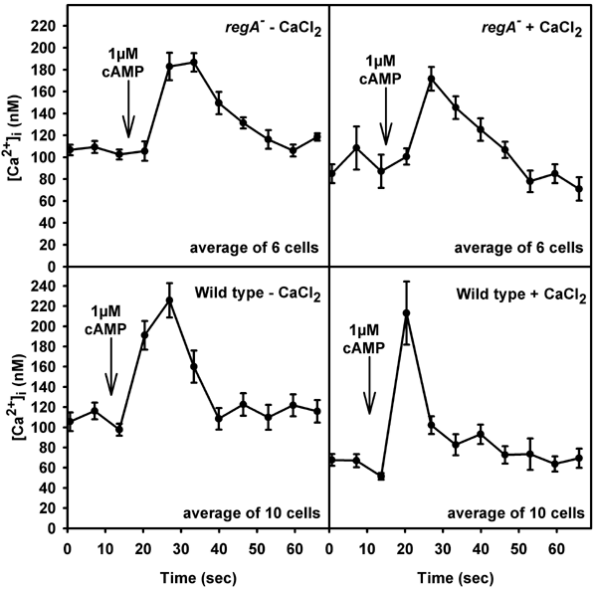
Measurement of cAMP activated [Ca^2+^]_i_-transients in wild type and mutant amoebae in the cytosolic presence of IBMX. IBMX led to an elevation of basal [Ca^2+^]_i_. Upon stimulation with 1 μM cAMP in the absence of external CaCl_2 _a [Ca^2+^]_i_-transient was observed in both, mutant and wild type amoebae (mean ± s.e.m.).

IBMX does not inhibit extracellular PDE [15] but affects cAMP hydrolysis intracellularly, so we compared basal [Ca^2+^]_i _and cAMP-activated [Ca^2+^]_i_-changes of *regA*^- ^to wild type cells in the absence and intracellular presence of IBMX. The inhibitor should affect the activity of both cAMP phosphodiesterases, RegA and PDE-E [16,17]. Without IBMX, basal [Ca^2+^]_i _was similar in both strains (Table 1). However, cAMP-addition induced a [Ca^2+^]_i_-transient in *regA*^- ^cells in nominally Ca^2+^-free medium (Fig. 2, Table 1). In wild type, cAMP-activated [Ca^2+^]_i_-changes were observed after preincubation with 1 mM Ca^2+ ^for 10–15 min only (see also [18]). After loading of IBMX into the cytosol both, basal [Ca^2+^]_i _and cAMP-induced [Ca^2+^]_i_-changes were altered. Basal [Ca^2+^]_i _in the presence and absence of extracellular Ca^2+ ^was significantly increased in *regA*^-^; the height of the [Ca^2+^]_i_-transient after cAMP-stimulation was comparable to the control situation. In wild type, basal [Ca^2+^]_i _was elevated and a [Ca^2+^]_i_-change was also observed after cAMP addition in nominally Ca^2+^-free medium (Fig. 3, Table 1). In summary, increasing cAMP levels augmented cAMP-induced [Ca^2+^]_i_-transients at concomitantly reduced levels of Ca^2+^-influx; the increase in basal intracellular cAMP caused by the absence of RegA was sufficient. Alteration of basal [Ca^2+^]_i _required an even higher concentration of cAMP. This was achieved by inhibition of RegA and of PDE-E via loading of IBMX into the cytosol. In wild type where both enzymes are present basal [Ca^2+^]_i _was not elevated in the presence of external Ca^2+ ^which indicates that the amount of cAMP had just reached a threshold value and that basal [Ca^2+^]_i _is more tightly controlled than agonist activated [Ca^2+^]_i_-changes.

**Table 1:** Basal [Ca^2+^]_i _and the increase over basal [Ca^2+^]_i _after cAMP-addition in wild type and *regA*^- ^cells in the absence and presence of IBMX. 1 μM cAMP was added to wild type at t_7_–t_8 _and to *regA*^- ^at t_4 _because the mutant develops more rapidly. [Ca^2+^]_i _was determined by ratiometric imaging in single cells either in nominally Ca^2+^-free buffer (- Ca^2+^) or in buffer containing 1 mM Ca^2+^. Values are mean ± s.e.m. and numbers in brackets indicate the numbers of cells tested in at least 3 determinations in at least 2 independent experiments each.

Strain Condition	Basal [Ca^2+^]_i_		cAMP-induced [Ca^2+^]_i_-change	
	- IBMX	+ IBMX	- IBMX	+ IBMX
regA^-^				
- Ca^2+^	55 ± 1 (131)	97 ± 1 (111)	71 ± 8 (30)	81 ± 9 (25)
+ 1 mM Ca^2+^	54 ± 1 (85)	96 ± 2 (66)	79 ± 6 (52)	85 ± 8 (35)
				
Wild type				
- Ca^2+^	53 ± 1 (94)	98 ± 1 (148)	no increase	132 ± 9 (58)
+ 1 mM Ca^2+^	50 ± 1 (185)	53 ± 1 (127)	125 ± 6 (83)	155 ± 10 (55)

**Figure 4:**
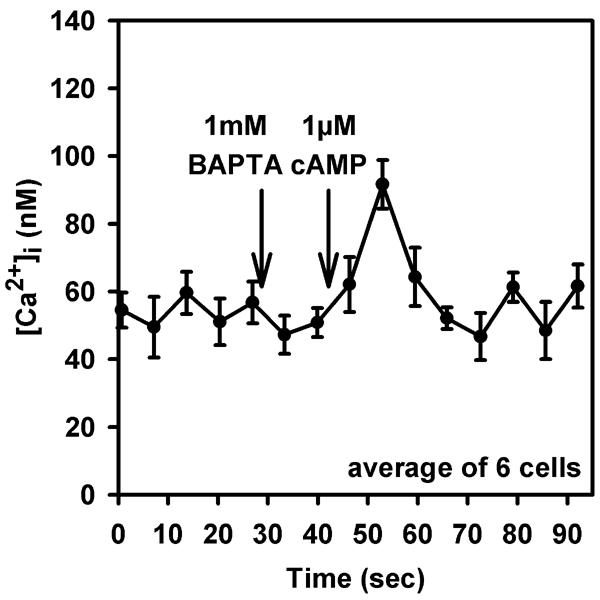
[Ca^2+^]_i_-changes in *regA*^- ^cells in the presence of a Ca^2+^-chelator. Amoebae in nominally Ca^2+^-free medium were challenged with 1 mM BAPTA (final concentration) and subsequently with 1 μM cAMP. Arrows indicate the time point of addition of agents. The graph shows the average increase (mean ± s.e.m.).

The effect of the increased basal cAMP concentration on the [Ca^2+^]_i_-regulation in *regA*^- ^amoebae might be caused by a change in the characteristics of Ca^2+^-fluxes of internal stores. A positive influence of cAMP via PKA-mediated phosphorylation of both, IP_3_-receptors and ryanodine receptors on release of stored Ca^2+ ^has been reported (for review see [19]). We therefore tested the response of *regA*^- ^amoebae upon stimulation with cAMP in the presence of the chelator BAPTA. We found that even after the addition of 1 mM BAPTA cAMP activated a transient increase in [Ca^2+^]_i _(Fig. 4). The elevation was smaller than that observed in nominally Ca^2+^-free medium and amounted to an average of 44 ± 3 nM above basal [Ca^2+^]_i _(mean ± s.e.m. of 18 determinations in 2 independent experiments). In wild type amoebae a cAMP-stimulated [Ca^2+^]_i_-increase is not detectable in the presence of BAPTA; the occurrence of a transient [Ca^2+^]_i_-elevation in *regA*^- ^cells indicates an augmented release of Ca^2+ ^from stores in the mutant. Support for an effect of cAMP via PKA came from experiments where we incubated cells with the membrane permeant activator of PKA, Sp-5,6-DCl-cBIMPS [20,21]. Basal [Ca^2+^]_i _was increased in *regA*^- ^cells upon treatment with 30 μM Sp-5,6-DCl-cBIMPS for 60 min (139 ± 2 nM; mean ± s.e.m. of 15 determinations in 3 independent experiments); agonist-induced [Ca^2+^]_i_-transients in nominally free Ca^2+^-buffer were unaltered in height (87 ± 8 nM; mean ± s.e.m.) as compared to control cells. In addition, we found that preincubation of wild type amoebae with 30 μM Sp-5,6-DCl-cBIMPS reduced cAMP-activated Ca^2+^-influx in cell suspensions by 26 ± 8% (mean ± s.e.m. of 3 independent experiments).

### Light scattering and extracellular Ca^2+ ^oscillations depend on internal cAMP levels

**Figure 5:**
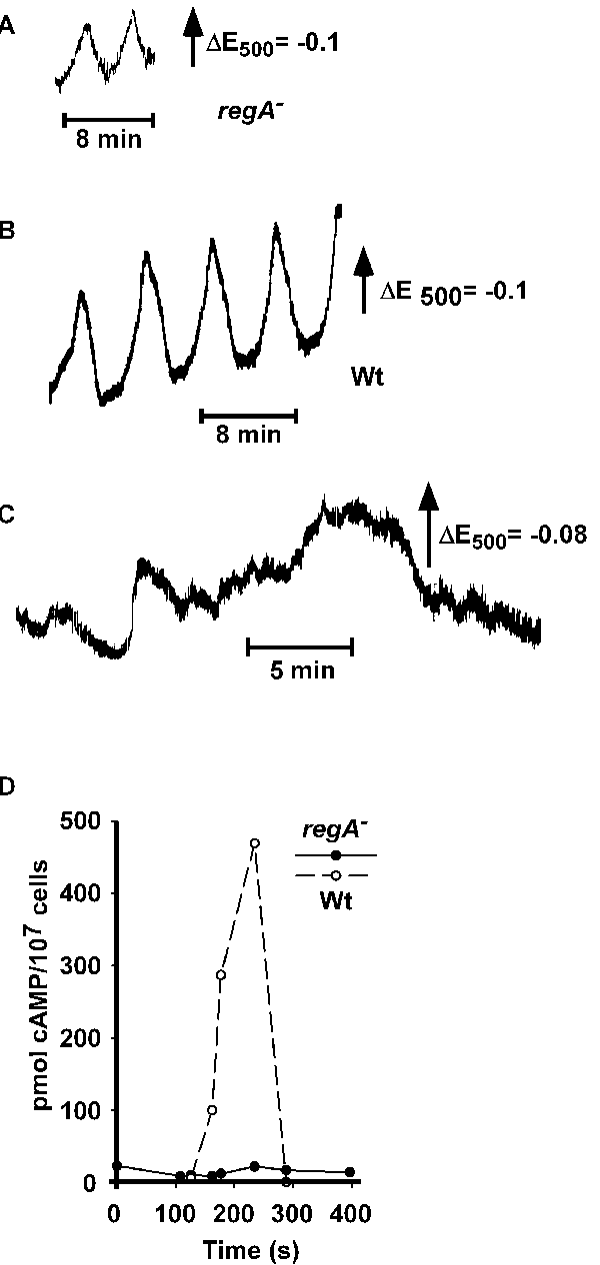
Light scattering and [Ca^2+^]_e _oscillations of *regA*^- ^cells. Light scattering and [Ca^2+^]_e _were recorded as outlined in Methods. (a, b) Regular light scattering oscillations with a phase length of roughly 4–5 min but with strongly reduced amplitude as compared to wild type oscillations (see also [3]). (c) Irregular light scattering changes. (d) Oscillations of cAMP levels in the *regA*^- ^strain were less pronounced than in the wild type; the graph shows examples of one cAMP oscillation each, determined during one spike of light scattering oscillations.

**Figure 6:**
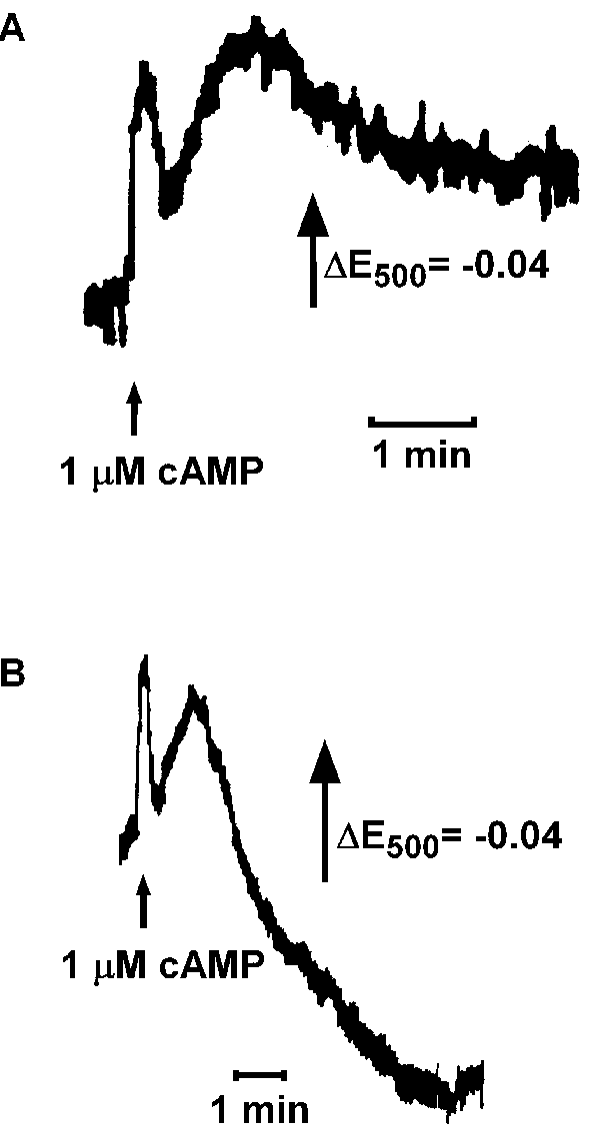
Light scattering response upon addition of 1 μM cAMP. (a) Wild type cells displayed two peaks of light scattering which subsequently returned to the baseline. (b) In *regA*^- ^cells there was a strong decrease in light scattering after the second peak. One out of 7 independent experiments is shown.

**Figure 7:**
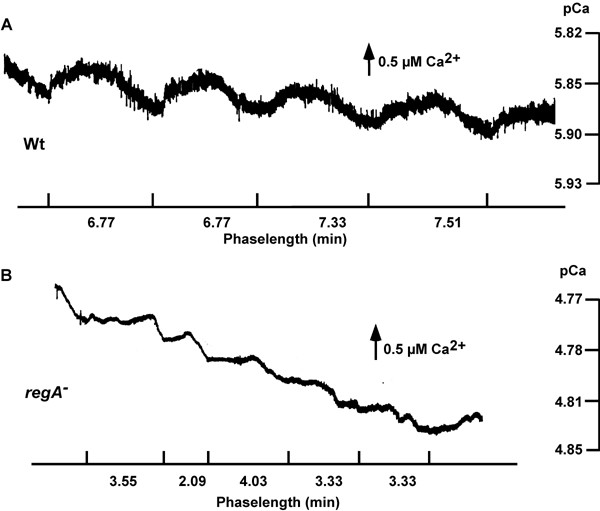
[Ca^2+^]_e _oscillations in wild type and *regA*^- ^cell suspensions. (a) Regular [Ca^2+^]_e _oscillations were recorded in wild type cell suspensions (see also [2]). (b) Similar to light scattering oscillations the pattern of [Ca^2+^]_e _oscillations in *regA*^- ^was irregular. One out of 5 independent experiments is shown.

We had shown previously that artificial changes of [Ca^2+^]_i_, either by affecting Ca^2+^-stores or by activating Ca^2+^-influx alter light scattering oscillations [3,4]. When light scattering was analyzed in *regA*^- ^suspensions two types of responses were observed. On one hand, regular oscillations with a phase length of 4.3 ± 1 min (mean ± s.d. of 61 determinations in 6 independent experiments) occurred (Fig. 5A). The amplitude of these oscillations was reduced as compared to wild type (Fig. 5B), i.e. by 78%. On the other hand, irregular light scattering changes were detected (Fig. 5C). Determination of cAMP levels revealed that cAMP scarcely oscillated in *regA*^- ^(Fig. 5D) and increased on average by a factor of 2.9 ± 0.6 (mean ± s.e.m. of 5 independent experiments). The response upon addition of cAMP was also different: after an increased first light scattering peak and the occurrence of a second peak light scattering did not return to the baseline as in wild type suspensions but fell well below (Fig. 6). The alteration in light scattering responses in the mutant might be due to a shift in sensitivity to cAMP. As a control we tested the reaction upon stimulation with cAMP and found that *regA*^- ^cells reacted when 3 nM cAMP was added (not shown) which indicates that the mutant strain is practically as sensitive as wild type. Measurement of [Ca^2+^]_e _in *regA*^- ^cell suspensions revealed irregular [Ca^2+^]_e _oscillations, similar to the results obtained for light scattering (Fig. 7).

### Chemotaxis of *regA*^- ^amoebae

**Figure 8:**
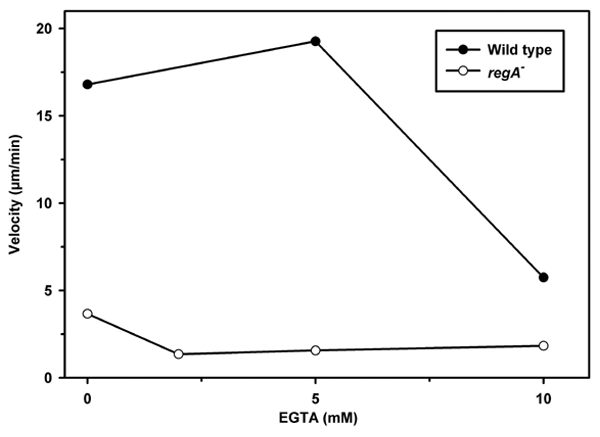
Chemotactic speed of wild type and *regA*^- ^amoebae. The effect of preincubation with EGTA for 30 min was assayed. Chemotactic velocity of amoebae was affected dose dependently by EGTA treatment; when compared to the wild type the speed of the *regA*^- ^strain was significantly reduced at lower concentrations of EGTA. Velocity of wild type and *regA*^- ^cells is shown (median of at least 2 independent experiments).

It had been reported previously that *regA*^- ^cells have a reduced capacity to suppress lateral pseudopod formation [14]. In accordance with the data presented by Wessels et al. [14] we also observed augmented lateral pseudopod extension upon challenge of aggregation competent amoebae with a cAMP filled glass capillary (not shown). The reduction in chemotactic polarization was reflected by a decrease in the average chemotactic speed as compared to wild type amoebae (Fig. 8). Pretreatment with EGTA to empty Ca^2+^-storage compartments dose-dependently inhibited chemotaxis of *regA*^- ^and wild type. The EGTA-incubated cells were rounded and extended only small pseudopods towards the capillary tip (not shown); in both strains chemotactic velocity was reduced. The effect was more pronounced in *regA*^-^: already in the presence of 2 mM EGTA cells chemotaxed more slowly than under control conditions (velocity of EGTA-treated amoebae was significantly lower at all concentrations of EGTA tested (P < 0.001) as compared to control cells; Mann Whitney rank sum test). Wild type cells were unaffected by preincubation with 5 mM EGTA for up to 1 hour whereas at 10 mM EGTA chemotaxis was reduced.

## Discussion

The cytosolic concentration of Ca^2+ ^was demonstrated to control light scattering oscillations by affecting the synthesis of cAMP; depending on the height of an artificial [Ca^2+^]_i_-transient the production of cAMP which in this case serves as first messenger was either augmented or blocked [3,4]. The results presented in this study provide evidence for a reciprocal influence of the second messengers cAMP and Ca^2+ ^in *Dictyostelium *cells. We observed altered agonist-induced Ca^2+^-fluxes and [Ca^2+^]_i_-transients in the *regA*^- ^mutant cell line where the absence of the main cAMP-hydrolyzing PDE led to a fourfold increased basal cAMP level. One could argue that the effect on [Ca^2+^]_i _was not a consequence of the increased basal concentration of cAMP but rather due to a potentially altered pattern of gene expression in the mutant strain. Indeed, this is possible and could result in a different signal perception and/or processing. However, we consider an alteration in gene expression unlikely to be responsible for the augmented [Ca^2+^]_i_-transients upon cAMP-stimulation since the same effect could be evoked in wild type amoebae by loading of the PDE inhibitor IBMX into the cytosol. In addition, IBMX evoked an increase in basal [Ca^2+^]_i _in both, wild type and mutant cells. In *regA*^- ^the inhibitor should act on the additional cAMP-PDE (PDE-E) [16,17] and therefore increase cAMP levels even further. In wild type amoebae hydrolysis of cAMP should be retarded as well. Yet, the threshold of the cAMP concentration required to increase basal [Ca^2+^]_i _might not be achieved as consistently as in the mutant since IBMX must act on both PDEs.

The sensitizing effect of the increased amount of cAMP on [Ca^2+^]_i _could be caused by several factors. Ca^2+^-flux characteristics can be changed by influencing Ca^2+^-channels and/or Ca^2+^-ATPases located on both, the plasma membrane and membranes of internal stores. When we analyzed Ca^2+^-fluxes with a Ca^2+^-sensitive electrode influx was reduced in the mutant while the rates of influx and efflux were unchanged. If the activity of the plasma membrane Ca^2+^-ATPase (PMCA) was altered then flux rates should be affected. Moreover, the reduced amount of Ca^2+^-influx precludes activation of a plasma membrane Ca^2+^-channel. In other cell systems activation of the PMCA and of Ca^2+^-channels by an increase in cAMP levels was shown [22-24] but our data argue against a stimulating effect on plasma membrane Ca^2+^-channel or PMCA activity in *Dictyostelium *amoebae.

The second target of action of cAMP are intracellular stores. Indeed, we showed for the first time that in *Dictyostelium *a cAMP-activated [Ca^2+^]_i_-elevation occurred in the extracellular presence of the Ca^2+^-chelator BAPTA. This argues for an alteration of Ca^2+^-uptake into and/or Ca^2+^-release from stores. An as yet unknown negative regulation of Ca^2+^-sequestration could cause accumulation of Ca^2+ ^in the cytosol; until now, however, activation of SERCA-type Ca^2+^-ATPases was found only (for review see [19]). On the other hand, release of Ca^2+ ^could have been augmented by the high basal cAMP level in the mutant. cAMP-dependent phosphorylation of the IP_3_-receptor by PKA results in increased sensitivity for IP_3 _in pancreatic acinar cells [25]; the same holds true for the ryanodine receptor [19]. Stimulation of PKA activity is plausible since pretreatment with the PKA-activator Sp-5,6-DCl-cBIMPS elevated basal [Ca^2+^]_i _and reduced agonist-evoked Ca^2+^-entry. Membrane permeable Sp-5,6-DCl-cBIMPS was shown to be virtually ineffective in inducing gene expression and to be highly selective for PKA vs cAMP receptor activation at the concentration employed [21]. In summary, we propose the following model: in the mutant sensitivity of the Ca^2+^-release system is enhanced by an augmented PKA-mediated phosphorylation which is due to increased basal cAMP levels. This results in larger amounts of Ca^2+ ^being liberated upon stimulation. In *Dictyostelium *release of Ca^2+ ^from stores was also found after addition of calmidazolium [26] which was shown to inhibit calmodulin-dependent and independent activity of calcineurin [27]. Calcineurin in turn was proposed to be responsible for termination of Ca^2+^-release by dephosphorylating the IP_3_-receptor [28]. In *regA*^- ^augmented release of Ca^2+ ^leads to a reduction of Ca^2+^-entry across the plasma membrane as a negative feedback.

We suggest the alteration in [Ca^2+^]_i _to be responsible for the irregular light scattering and extracellular [Ca^2+^]-oscillations of *regA*^- ^cells. Previously, Wessels et al. [14] have shown that the mutant cannot propagate a cAMP wave since wild type amoebae no longer aggregated correctly when mixed with mutant cells. Indeed, we found that peak cAMP levels during light scattering oscillations were very low in *regA*^- ^as compared to wild type. This effect is plausible, as the increased sensitivity of the Ca^2+ ^second messenger system exerts a negative feedback on cAMP synthesis: large [Ca^2+^]_i_-transients inhibit production of cAMP [3]. An interplay of cAMP and [Ca^2+^]_i_-oscillations and their mutual dependence has also been shown in neurons: absence of either, cAMP or [Ca^2+^]_i_-oscillations resulted in failure of the other component to oscillate [8]. In *Dictyostelium *the strong decrease in peak cAMP oscillation levels affected [Ca^2+^]_e_-oscillations which were irregular. The basis is probably an influence on [Ca^2+^]_i_-oscillations. Such oscillations were suggested to occur but have not been demonstrated in single cells until now, presumably due to the small size of the amoebae and the characteristics of the wave [29].

With respect to chemotaxis, reduced suppression of lateral pseudopod formation was shown in *regA*^- ^cells and an essential role of RegA for a correct response in a natural cAMP wave and chemotactic migration was assigned [14]; subsequently, a similar result was found in a mutant expressing a constitutively active PKA [30]. When we analyzed chemotaxis towards a cAMP-filled glass capillary we observed the same behaviour as described by Wessels et al. [14]. In principle, it is possible that the reduced capacity of *regA*^- ^cells to polarize was due to a difference in the developmental stage as compared to wild type cells. However, *regA*^- ^develops much faster than wild type which suggests an even more efficient chemotaxis as this response increases during differentiation to aggregation competence. Alternatively, an altered or dampened signaling response caused by a lower number of cAMP receptors present on the cell surface could have caused the reduced chemotactic response. We consider this to be unlikely for the following reason. Aggregation-competent *Dictyostelium *amoebae possess roughly 50.000 cAMP receptors at the cell surface [31]. Yet, for chemotactic orientation and polarization in a cAMP gradient the difference in receptor occupancy between the front and the rear end of the amoebae is important rather than the absolute number of stimulated receptors [31]. So even if *regA*^- ^expressed less receptors than wild type this should not influence the accuracy of the response. We propose the reduced polarization capacity of *regA*^- ^amoebae to be caused by their altered [Ca^2+^]_i_-regulation. In the mutant strain the threshold for generation of an agonist-induced [Ca^2+^]_i_-increase is lower than in wild type. The [Ca^2+^]_i_-elevation is not as tightly controlled and occurs even in the presence of BAPTA. The characteristics of a [Ca^2+^]_i_-increase are important for the resulting cytoskeletal rearrangements and whether pseudopods are formed correctly. Indeed, artificial induction of a small global [Ca^2+^]_i_-transient by incubation with calmidazolium caused overall pseudopod protrusion [26]. In migrating cells the establishment of a [Ca^2+^]_i_-gradient at the rear end was shown [5,32] which indicates the presence of a highly organized spatial [Ca^2+^]_i_-pattern during chemotaxis. By contrast, a role of the [Ca^2+^]_i_-elevation for the chemotactic response was questioned by Traynor et al. [33] because a mutant disrupted in a gene bearing similarity to IP_3_-receptors of higher eukaryotes aggregated and differentiated almost normally but displayed no cAMP-activated global [Ca^2+^]_i_-change; yet, the existence of localized, small [Ca^2+^]_i_-transients in this particular mutant cell line that had escaped detection could not be excluded [33].

When we analyzed the influence of pretreatment with EGTA on chemotactic behaviour of wild type and *regA*^- ^cells we found that the mutant was more sensitive. When compared to wild type, lower doses of EGTA were sufficient to reduce chemotactic speed. The effect of EGTA treatment is most probably due to emptying of the storage compartments [34]; the presence or absence of extracellular Ca^2+ ^affects the Ca^2+^-content of stores [35,36]. *RegA*^- ^cells are more sensitive than wild type amoebae because of the lower threshold for Ca^2+ ^release and thus a more rapid depletion of Ca^2+ ^in the cells.

## Conclusion

Abnormal basal levels of cAMP impair chemotactic performance by augmenting agonist-activated [Ca^2+^]_i_-elevations which in turn lead to uncontrolled pseudopod extension. [Ca^2+^]_i _regulates cAMP acting as first messenger in a negative feedback loop: when the [Ca^2+^]_i _response is increased the amount of cAMP synthesized upon stimulation is low as observed in *regA*^- ^cells devoid of the phosphodiesterase RegA. The low level of cAMP relay results in improper light scattering oscillations. We conclude that intracellular cAMP acts on [Ca^2+^]_i _via PKA: phosphorylation of the system responsible for release of Ca^2+ ^from stores leads to a greater sensitivity facilitating Ca^2+ ^liberation. The cAMP activated [Ca^2+^]_i_-increase is due to Ca^2+^-release from internal stores which triggers subsequent extracellular Ca^2+^-entry. The fraction of the [Ca^2+^]_i_-elevation that is mediated by liberation of Ca^2+ ^is thus larger in the mutant.

## Methods

### Materials

Fura2-dextran and BAPTA were from MoBiTec (Göttingen, FRG). IBMX was purchased from Sigma (Munich, FRG) and cAMP was from Boehringer (Mannheim, FRG). Sp-5,6-DCl-cBIMPS was from Biomol (Hamburg, FRG).

### Cell culture

*D. discoideum *axenic wild type Ax2 was grown as described [4]; the mutant *regA*^- ^(kindly provided by Dr. P. Thomason) was grown in the presence of blasticidinS. Cells were washed by repeated centrifugation and resuspension of the cell pellet in cold Sørensen phosphate buffer (17 mM Na^+^/K^+^-phosphate, pH 6.0; SP-buffer). Amoebae were shaken at 2 × 10^7 ^cells/ml, 150 rpm and 23°C until use. The time, in hours, after induction of development is designated t_x_.

### Recording of light scattering

At t_2.5_–t_4 _2 ml of cell suspension was pipetted into cuvettes and aerated. Light scattering oscillations were recorded at 500 nm with a photometer as described [4].

### Determination of cAMP

The total amount of cAMP was determined using the cAMP enzyme immuno assay (Biotrak, Amersham Pharmacia Biotech, Freiburg, FRG) according to the manufacturer's instructions. Samples were prepared as outlined previously [4].

### Extracellular [Ca^2+^]-measurements

The extracellular Ca^2+^-concentration ([Ca^2+^]_e_) was measured in 2 ml of cell suspension (5 × 10^7 ^cells/ml in 5 mM Tricine, 5 mM KCl, pH 7.0) with a Ca^2+^-sensitive electrode (Möller, Zürich, Switzerland) as described [18]. [Ca^2+^]_e_-oscillations were measured at a cell density of 1 × 10^8 ^cells/ml.

### Single cell [Ca^2+^]_i_-imaging

Cytosolic [Ca^2+^]-imaging was done as outlined in [6]. Cells (5 × 10^7 ^cells/ml; 20 μl) were loaded at t_3 _with the Ca^2+^-indicator fura2-dextran (concentration in the loading solution: 5 mg/ml SP-buffer + 1 mM CaCl_2_) by electroporation (0°C, 850 V, 3 μF, 200 Ω). Immediately after electroporation, 80 μl of cold 5 mM MgCl_2 _was added and cells were incubated for 10 min on ice. Then cells were washed 3× with 5 mM Hepes, pH 7.0 (H5-buffer). Washed cells (2–5 μl) were placed on glass coverslips and incubated in a humid chamber until use. When experiments were done in nominally Ca^2+^-free medium, 85–88 μl of H5-buffer was added 1 min before the [Ca^2+^]-imaging experiment. To test the response of amoebae in the presence of BAPTA, 75–78 μl of H5-buffer was pipetted to the cells; 10 μl of 10 mM BAPTA was added during the [Ca^2+^]-imaging experiment and 10–12 sec later cAMP was given. When the response of cells was to be analyzed in the presence of extracellular CaCl_2_, H5-buffer (85–88 μl) with 1 mM CaCl_2 _was added to the cells 15 min before the [Ca^2+^]-imaging experiment to load stores (see also [18]). cAMP-stimulation was done by adding 10 μl of 10 μM cAMP (± 1 mM CaCl_2_) to the cells. To load cells with IBMX, they were electroporated with fura2-dextran in the presence of 250 μM of the inhibitor. The cytosolic concentration of IBMX is in the range of maximally 2–5% of the concentration present during electroporation [6]. Measurement of *regA*^- ^was done at t_4 _and wild type [Ca^2+^]-imaging was done at t_7–8_. In another series of experiments we treated *regA*^- ^cells with Sp-5,6-DCl-cBIMPS, a membrane permeant activator of PKA [20]. Incubation was done with 37 μM of the activator for 60 min prior to the [Ca^2+^]-imaging experiment.

### Chemotaxis of *regA*^- ^cells

Chemotactic performance of the amoebae depends on the degree of differentiation, so their shape was checked prior to the chemotaxis assay. 200 μl of cells at 2 × 10^7 ^cells/ml were placed on a coverslip and allowed to settle for at least 30 min. The morphology of the cells was controlled microscopically: when elongated and thus aggregation competent cells were present, an aliquot of cells from the suspension was diluted for the chemotaxis assay. *RegA*^- ^was tested at t_4_–t_5_, wild type was measured at t_7_–t_10_. 250 μl of cells in 5 mM Hepes, pH 7.0 (1 × 10^5 ^cells/ml) were placed on glass coverslips. After 30 min cells were challenged with a cAMP (100 μM) filled glass capillary and chemotaxis was recorded for 40–45 min either on vidoe tape or images were stored directly on a hard disk. In addition, experiments were done with cells incubated with 2–10 mM EGTA for 30 min to empty Ca^2+^-storage compartments. Analysis of chemotaxis was done as outlined previously [34].

## List of abbreviations

Cytosolic free Ca^2+ ^concentration: [Ca^2+^]_i_

Phosphodiesterase: PDE

3-isobutyl-1-methylxanthine: IBMX

cAMP-dependent protein kinase: PKA

Plasma membrane Ca^2+^-ATPase: PMCA

## Authors' contributions

DFL performed extracellular [Ca^2+^] recordings and light scattering experiments. He also determined cAMP levels and designed the study. KBR did chemotaxis experiments at different external conditions. KH carried out [Ca^2+^]_i_-measurements. CS did [Ca^2+^]_i_-imaging experiments, designed the study and wrote the manuscript. All authors read and approved the manuscript.
